# Accurate prediction of major histocompatibility complex class II epitopes by sparse representation via ***ℓ***_1_-minimization

**DOI:** 10.1186/1756-0381-7-23

**Published:** 2014-11-04

**Authors:** Clemente Aguilar-Bonavides, Reinaldo Sanchez-Arias, Cristina Lanzas

**Affiliations:** 1 National Institute for Mathematical and Biological Synthesis, University of Tennessee, 37996-3410 Knoxville, TN, USA; 2 Department of Applied Mathematics, Wentworth Institute of Technology, 02115 Boston, MA, USA; 3 Department of Biomedical and Diagnostic Sciences, University of Tennessee, 37996-3410 Knoxville, TN, USA

**Keywords:** Peptide binding, Sparse representation, MHC class II, Epitope prediction, Immunoinformatics, Machine learning, Classification algorithms

## Abstract

**Background:**

The major histocompatibility complex (MHC) is responsible for presenting antigens (epitopes) on the surface of antigen-presenting cells (APCs). When pathogen-derived epitopes are presented by MHC class II on an APC surface, T cells may be able to trigger an specific immune response. Prediction of MHC-II epitopes is particularly challenging because the open binding cleft of the MHC-II molecule allows epitopes to bind beyond the peptide binding groove; therefore, the molecule is capable of accommodating peptides of variable length. Among the methods proposed to predict MHC-II epitopes, artificial neural networks (ANNs) and support vector machines (SVMs) are the most effective methods. We propose a novel classification algorithm to predict MHC-II called sparse representation via *ℓ*_1_-minimization.

**Results:**

We obtained a collection of experimentally confirmed MHC-II epitopes from the Immune Epitope Database and Analysis Resource (IEDB) and applied our *ℓ*_1_-minimization algorithm. To benchmark the performance of our proposed algorithm, we compared our predictions against a SVM classifier. We measured sensitivity, specificity abd accuracy; then we used Receiver Operating Characteristic (ROC) analysis to evaluate the performance of our method. The prediction performance of MHC-II epitopes of the *ℓ*_1_-minimization algorithm was generally comparable and, in some cases, superior to the standard SVM classification method and overcame the lack of robustness of other methods with respect to outliers. While our method consistently favoured DPPS encoding with the alleles tested, SVM showed a slightly better accuracy when “11-factor” encoding was used.

**Conclusions:**

*ℓ*_1_-minimization has similar accuracy than SVM, and has additional advantages, such as overcoming the lack of robustness with respect to outliers. With *ℓ*_1_-minimization no model selection dependency is involved.

## Background

Pathogen peptide fragments are displayed on the surface of professional antigen presenting cells via the Major Histocompatibility Complex (MHC) class II. Such peptide fragments are known as epitopes. When helper T cells recognize epitopes bound to MHC-II, an adaptive immune response can be triggered for a specific pathogen. Computational prediction of MHC-II binding peptides can accelerate the development of vaccines and immunotherapies by identifying a narrow set of epitope candidates for further testing. Prediction of MHC-II epitopes is particularly challenging because the open binding cleft of the MHC-II molecule allows epitopes to bind beyond the peptide binding groove; therefore, the molecule is capable of accommodating peptides of variable length [1]. The binding core of the MHC-II is approximately nine amino acids long [2]; however, the complete epitope length can vary from 9 to 25 amino acids [3] and may even bind to whole proteins [4]. In addition, a successful computational prediction is based on sufficiently large set of high quality training data. Obtaining a large dataset for MHC-II epitope prediction can be difficult.

Machine-learning methods like artificial neural networks (ANNs) and support vector machines (SVMs) are classification techniques that have been successfully applied to predict MHC-II binding [5,6]. These methods, however, have some limitations. The biggest limitation of SVM lies in the choice of the kernel. The best choice of kernel for a given problem is still a research problem [7]. SVMs deliver a unique solution because the optimality problem is convex. On the other hand, ANNs have multiple solutions associated with local minima, which makes the method unrobust. The sparse representation (SR) approach proposed in this paper for peptide binding classification relies on the natural selective nature of the solution of an *ℓ*_1_-minimization problem [8]. This method overcomes the limitations of the machine-learning methods. Model selection is not necessary to differentiate between two classes; this contrasts with the need to test different kernel functions in the SVM approach when trying to find the best separating hyperplane with larger margin between classes. Furthermore, the use of the *ℓ*_1_ norm promotes robustness in the method with respect to outliers in the data being used for classification [8], discarding bad training samples and allowing the handling of noisy data.

The *ℓ*_1_ norm of a vector $x\in R^{n}$ is defined as the sum of the absolute values of each of its components, i.e.,

(1) $$∥x{∥}_{1}=|x_{1}|+|x_{2}|+\ldots +|x_{n}|.$$

Convex relaxation approaches based on the *ℓ*_1_ norm have been proven to successfully promote sparse solutions (i.e. solutions with few nonzero elements) to linear system of equations with high probability. The work in the area of compressed sensing initiated in late 2004 by Emmanuel Candés, Justin Romberg and Terence Tao, and independently by David Donoho [8] encouraged the implementation of different fast solvers capable to find sparse solutions using the *ℓ*_1_ norm as regularizer. Applications in science and technology have been successfully implemented with promising results in signal reconstruction, image processing, inverse problems, data analysis, among others. Finding sparse solutions has brought practical benefits such as the need of fewer antennas for remote sensing, fewer measurements needed in geophysical surveys, and more precise identification of genes.

The goal of this project is twofold. First, to develop a classifier using the SR approach for epitopes of variable length with the largest margin between the observations belonging to two different classes (binders/non-binders), while minimizing the training error. Second, to evaluate epitope encoding techniques for binding prediction.

## Methods

MHC molecules are extremely polymorphic, with different alleles and thousands of epitopes identified in humans and other vertebrates [9]. To have a varied testing data set in terms of alleles and number of entries, we selected two different alleles for mice (H2-IA^b^ and H2-IA^d^) and three alleles for humans (HLA-DRB1*0101, HLA-DPA1*0103/ DPB1*02:01 and HLA-DRB1*0401). These alleles have been used previously in computational experiments [5,9,10]. Peptide sequences and their binding affinities from the alleles selected were collected from the Immune Epitope Database and Analysis Resource (IEDB) [11] (Table 1). This database contains data related to antibody and T cell epitopes for humans, non-human primates, rodents, and other animal species. We removed duplicated epitopes and unnatural peptides with more than 75% alanine. To further evaluate the prediction performance and robustness of our algorithm we generated receiver Operating Characteristic (ROC) curves, distinguishing binders and non-binders and taking into consideration different cut-off points according to the half maximal inhibitory concentration (*IC*_50_) for each epitope, as shown in Table 1.

**Table 1:** Peptide sequences and their binding affinities

	***I*** ***C***_**50 **_**cuttoff**	**100**	**300**	**500**	**1000**	**5000**	**10000**	**15000**
	Bind	49	93	112	153	233	285	321
H2-IA^b^	Non-bind	485	441	422	381	301	249	213
	Total	534	534	534	534	534	534	534
	Bind	34	43	52	56	68	70	79
H2-IA^d^	Non-bind	214	205	196	192	180	178	169
	Total	248	248	248	248	248	248	248
HLA-DPA1*0103/	Bind	32	53	67	92	149	171	175
DPB1*0201	Non-bind	264	243	229	204	147	125	121
	Total	296	296	296	296	296	296	296
	Bind	2253	2988	3326	3799	4503	4783	4924
HLA-DRB1*0101	Non-bind	3095	2360	2022	1549	845	565	424
	Total	5348	5348	5348	5348	5348	5348	5348
	Bind	58	87	102	139	196	210	270
HLA-DRB1*0401	Non-bind	252	223	208	171	114	100	40
	Total	310	310	310	310	310	310	310

### Data

### Encoding scheme

The most common way of amino acid encoding is the binary encoding scheme represented by a 20-bit binary vector, where 19 bits are set to zero and one bit is set to 1. Property encoding, on the other hand, is based on a vector containing one or more amino acid properties. Property encoding has two main advantages over binary encoding. First, physicochemical properties play an important role in biomolecular recognition; therefore, this type of encoding is more informative. Secondly, property encoding mitigates the problem of flexible lengths. To test the reliability of property encoding, we used classical binary encoding and compared it against two property encoding methods, 11-factor encoding and divided physicochemical property scores (DPPS). The 11-factor encoding is calculated from physicochemical properties of amino acids as described by [12]. The properties were obtained from general physicochemical properties of amino acids and a number of properties identified in 3-D quantitative structure-activity relationship (QSAR) analysis [13]. The DPPS scheme was proposed by [14]. The DPPS descriptor was obtained by applying principal component analysis (PCA) to thousands of amino acid structural and property parameters. The resulting transformation yielded score vectors involving significant nonbinding properties of each of the 20 amino acids.

We represented every epitope of length *n* as a vector of 10 or 11 factors, corresponding to second and third encoding schemes, respectively, by adding to each position of the vector *v*_*i*_ the amino acid *x*_*i*_ 11-factor encoding or DPPS values in the following way:

(2) $$v_{i}=\overset{n}{\underset{i=1}{\sum }}x_{i},$$

Thus, every vector correlates directly with the physicochemical properties of amino acids, allowing the prediction of class II-peptide interaction. Additionally, we mitigated the problem of flexible lengths since every epitope is represented as a vector of size 10 or 11.

### Classification via sparse representation

We applied the *selective nature* of sparse representation to perform classification. As presented in [8], *ℓ*_1_-minimization techniques provide a satisfactory method to solve sparse representation problems. We propose a classifier based on the solution of an *ℓ*_1_-minimization problem for classification. A supervised learning system performing classification is commonly called a *classifier*.

Formally, given an input dataset, **W **= {**w**_1_,…,**w**_*n*_}, a set of labels/classes **T **= {*t*_1_,…,*t*_*n*_}, and a training dataset **D **= {(**x**_*i*_,*t*_*i*_):*i *= 1,…,*n*}, such that *t*_*i*_ is the label/class associated to the sample **x**_*i*_, a classifier is a mapping  from **W** to **T**, assigning the correct label *t *∈ **T** to a given input **w **∈ **W**, that is, F(w,D)=t.

Let us consider a *training data set*$\{(x_{i},t_{i}):i=1,\ldots ,n\},\;x_{i}\in R^{d},\;t_{i}\in \{1,2,\ldots ,N\},$ where *n* is the number of samples and *N* the number of classes. The vector $x_{i}\in R^{d},$ represents the *i*th sample (for instance containing “gene expression” values, special features, etc), and *t*_*i*_ denotes its corresponding label (in our case, binding or non-binding). Assume that *d *< *n*, that is, the length of each sample is less than the number of elements in the training dataset.

The sparse representation problem is formulated as follows: For a testing sample $y\in R^{d},$ find the sparsest vector **c **= [ *c*_1_,*c*_2_,…,*c*_*n*_]^*T*^ such that

(3) $$y=c_{1}x_{1}+c_{2}x_{2}+\ldots +c_{n}x_{n}.$$

Equation (3) states that we express the vector *y* as a linear combination of the collection {**x**_1_, **x**_2_,…,**x**_*n*_}. Using matrix algebra notation, equation (3) can be posed as the underdetermined linear system of equations

(4) y=Ac,

where the matrix $A\in R^{n\times d}$ is constructed such that the *j*th column corresponds to sample **x**_*j*_, and the vector **c **= (*c*_1_,…,*c*_*n*_)^*T*^. Since we look for a sparse vector **c**, equation (3) states that the test sample **y** is a *linear combination of only a few training samples*. We are interested in the sparsest solution of the system of linear equations in (4). In order to find such a sparse solution, we solve the following *ℓ*_1_-optimization problem

(5) $$\begin{array}{l} \;\underset{c,e}{\text{min}}\;\lambda ∥c{∥}_{1}+\frac{1}{2}e^{T}e\\ \text{subject to}\;Ac-e=y, \end{array}$$

In [8], a novel optimization algorithm was proposed to solve problem (5) based on a iterative smooth convex relaxation methodology. One of the advantages of our formulation is that lack of robustness with respect to noise, missing data, and outliers can be overcome (a known property of the *ℓ*_1_ norm is the regularization of an inverse problem). An additional advantage is that we do not need to care for model selection because the selective nature of the sparse representation captures the level of membership of a given input in one of the different classes. In the following section, we describe how to decide the class of a given input after obtaining its sparse representation. The approach consists of associating the nonzero components of **c** with the columns of *A* corresponding to those training samples that have the same class. First, let *Ω*_*k*_ denote the set of indices given by

(6) $$\Omega_{k}=\left\{j\;:\;\text{training sample}\;x_{j}\;\text{has label}\;t_{j}=k\right\}.$$

Therefore,

(7) $$\Omega_{1}\cdot \cup \Omega_{2}\cdot \cup \ldots \cdot \cup \Omega_{N}=\left\{1,2,\ldots ,n\right\},$$

that is, the collection of sets of indices ${\left\{\Omega_{i}\right\}}_{i=1}^{N}$ forms a partition of the set {1,2,…,*n*}, where *n* is the amount of samples available in the training dataset.

Then we define the *discriminant* functions by

(8) $$g_{k}(y)=∥y-Ac_{k}{∥}_{2},\;k=1,\ldots ,N,$$

where *A***c**_*k*_ is defined by

(9) $$Ac_{k}=\underset{j\in \Omega_{k}}{\sum }c_{j}x_{j},$$

Notice that the function *g*_*k*_ in (8) measures the error obtained when the testing sample **y** is represented with elements of the training set that have the same class *k*. Finally, we classify **y** in the category with the smallest approximation error. That is, we compute

(10) $$\begin{array}{ll} g_{s}(y) & =\text{min}\left\{g_{1}(y),\;g_{2}(y),\ldots ,\;g_{N}(y)\right\},\\ \;t & =s, \end{array}$$

and conclude that the testing sample **y** has label **t**=*s*. In this manner, we identify the class of the test sample **y** based on how effectively the coefficients associated with the training samples of each class recreate **y**. 

### Support vector machines (SVM)

We compared the results of our proposed method for classification problems with the well known SVM strategy that has been commonly used in different pattern recognition and machine learning applications. SVMs are a set of related supervised learning methods that analyze data and recognize patterns commonly used for classification and regression analysis. The original SVM algorithm was proposed by Vladimir Vapnik and the current standard implementation was proposed by Corinna Cortes and Vladimir Vapnik, [15]. Standard SVM takes a set of input data and predicts for each given input which of two possible classes the input is a member of, which makes the SVM a non-probabilistic binary linear classifier. Intuitively, an SVM model is a representation of the samples as points in space, mapped so that the samples of the separate categories are divided by a clear gap that is as wide as possible.

Slow training is a possible drawback of SVM approaches because SVMs are trained by solving quadratic programming problems where the number of variables is equal to the number of samples in the training data set. When a large number of training data is available, the training process might turn slow. More information about the different strategies used in SVM for classification problems are described in [16]. Here we use the implementation of SVM available in MATLAB as part of the Statistics Toolbox, and report the results for the best possible setup (using radial basis functions) found after an appropriate parameter tuning stage (model selection).

### Evaluation of method performance

**Figure 1:**
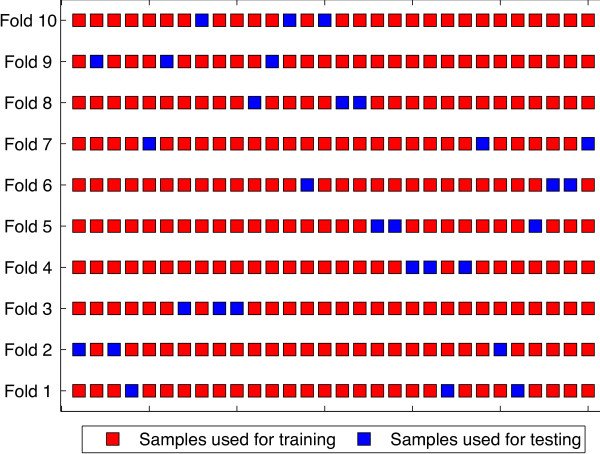
A 10-fold cross validation partition example.

To evaluate the prediction performance and robustness of our algorithm, we performed a 10-fold (*n*-fold) cross-validation. An illustration of the 10-fold cross validation partition process is shown in Figure 1. In the *n*-fold cross-validation, all the binding and non-binding epitopes were mixed and then divided equally into *n* parts, keeping the same distribution of binders and non-binders in each part. Then *n*-1 parts were merged into a training data set while the remnant was taken as a testing data set. This process was repeated 10 times and the average performance of *n*-fold cross-validation computed. We then measured sensitivity (Sn), specificity (Sp), accuracy (Acc) and Matthew’s Correlation Coefficient (MCC) for every fold and then took the average (Avg) as shown in Table 2. In addition, we performed a ROC curves analysis using different *IC*_50_ thresholds.

**Table 2:** **Average results for 10-fold cross-validation with *ℓ***_**1**_**-minimization and SVM**

			***ℓ***_**1**_**-minimization**			
		***H2-IA***^***d***^			HLA-DRB1*0101	
	**DPPS**	**11-factor**	**Binary**	**DPPS**	**11-factor**	**Binary**
Avg. Sn (*%*)	89 (6)	86 (2)	81 (12)	97 (6)	97 (25)	97 (22)
Avg. Sp (*%*)	86 (10)	54 (9)	82 (10)	29 (10)	8 (22)	9 (21)
Avg. Acc. (*%*)	88 (4)	70 (7)	82 (8)	85 (8)	82 (12)	82 (15)
Avg. MCC	0.7558	0.4418	0.6441	0.3715	0.1	0
Time in secs	0.1650	0.1368	0.1368	1.5198	1.6831	1.9103
			**SVM**			
Avg. Sn (*%*)	78 (4)	81 (1)	78 (1)	83 (7)	97 (23)	100 (37)
Avg. Sp (*%*)	76 (10)	76 (2)	77 (6)	72 (12)	31 (20)	2 (37)
Avg. Acc. (*%*)	77 (3)	78 (6)	77 (8)	81 (7)	86 (10)	83 (19)
Avg. MCC	0.5559	0.5804	0.5694	0.4738	0.3951	0
Time in secs	0.1039	0.1268	0.1597	0.1770	0.2045	0.1928

Numbers in parenthesis indicate standard deviation.

We examined the association between cutoff value, encoding factor and method for every allele, after 10-fold cross-validation using logistic regression analysis. The results show that sensitivity and specificity are statistically associated with the three predictors in most cases, whereas no significant associations were seen in accuracy. Results shown in Table 3.

**Table 3:** Logistic regression p-values with cutoff value, encoding factor and predictive method as predictors

		**Cutoff**	**Encoding**	**Method**
***H2-IA***^***b***^	Sn	***	***	*
	Sp	***	***	***
	Acc	***	NS	NS
***H2-IA***^***d***^	Sn	NS	NS	***
	Sp	***	***	***
	Acc	NS	NS	NS
HLA-DPA1*0103/DPB1-0201	Sn	***	***	***
	Sp	***	***	***
	Acc	NS	NS	NS
HLA-DRB1*0101	Sn	***	***	**
	Sp	***	***	**
	Acc	***	NS	NS
HLA-DRB1*0401	Sn	***	***	**
	Sp	***	***	***
	Acc	NS	NS	NS

95% Confidence interval (CI) and P-values NS (No significant), *P < 0.05, **P < 0.01, ***P <.001.

## Results

### Prediction accuracy

Techniques for predicting MHC binding include ANNs (NetMHCpan [6] and NN-Align [17]), position Specific Scoring Matrices (PSSMs) (RANKPEP [18]), and amino acid pairwise contact potentials as input vector for SVM (EpicCapo [10]). These methods have typical prediction accuracies of almost 70-90*%*[19]. Overall, our binding prediction accuracies are comparable to the reported 70-90*%* accuracies. Table 2 shows a comparison of the three encoding schemes with two alleles. While our method consistently favors DPPS encoding with the alleles tested, SVM shows a slightly better accuracy with 11-factor encoding. The experiments performed indicate that the physicochemical properties of amino acids are more informative in predicting MHC-II binding peptides. These results are also consistent with the MCC obtained for binary encoding, which yielded negative and zero scores on various occasions, implying that the predictions were not better than random predictions.

### ROC curves analysis

We also applied ROC analysis to examine the performance of the *ℓ*_1_-minimization and SVM classifiers. An ROC graph is a plot with the false positive rate on the *x* axis (1 - specificity) and the true positive rate on the *y* axis (sensitivity). The point (0,1) is the perfect classifier: it classifies all positive cases and negative cases correctly. The point (0,0) represents a classifier that predicts all cases to be negative, while the point (1,0) is the classifier that is incorrect for all classifications. The ROC curves were calculated using the thresholds shown in Table 1 to distinguish binders from non-binders.

**Figure 2:**
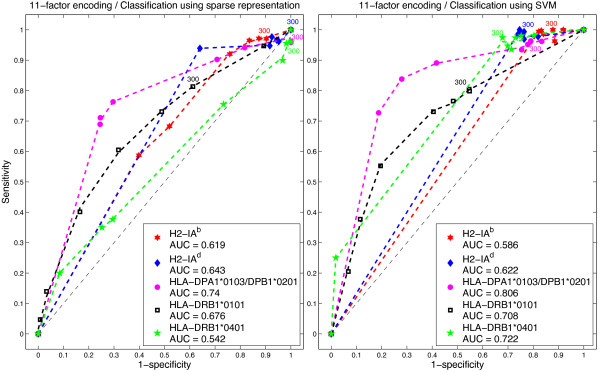
**11-factor encoding.** ROCs for sparse representation and SVM.

**Figure 3:**
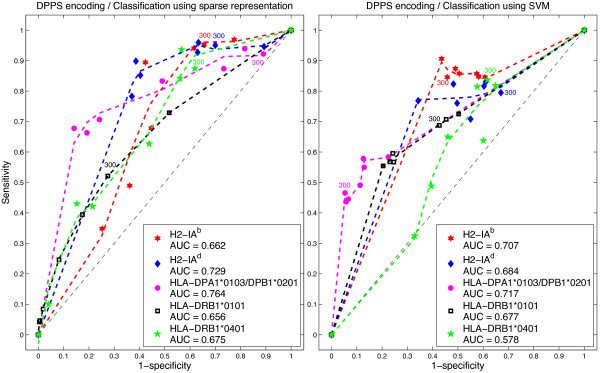
**DPPS encoding.** ROCs for sparse representation and SVM.

**Figure 4:**
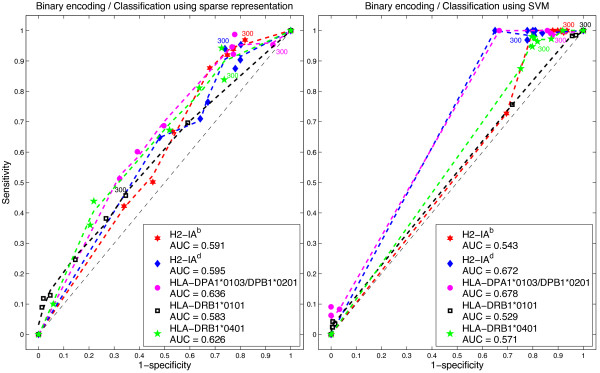
**Binary encoding.** ROCs for sparse representation and SVM.

In Figures 2, 3 and 4 we present the corresponding ROCs for the sparse representation method and ROCs were calculated using different IC _50_ cutoff values.The area under the ROC curve (AUC) provides a measure of overall prediction accuracy. An AUC value of 0.5 indicates random choice; while values close to 1 indicate excellent predictive capabilities of the method used. AUC values were computed using trapezoidal rule for numerical integration. With DPPS encoding, the *ℓ*_1_-minimization method for predicting epitopes on H2-IA^d^ and HLA-DPA1*0103/DPAB1*0201 molecules rendered AUC values of 0.729 and 0.764, respectively, higher than any of the AUC of SVM for the same encoding scheme. However, with 11-factor encoding, the AUC obtained by SVM was 0.806 for molecule HLA-DPA1*0103/DPAB1*0201, a higher value than any AUC obtained by *ℓ*_1_-minimization. Tables 4, 5, 6, 7 and 8 show the values of sensitivity (Sn), specificity (Sp) and accuracy (Acc) for each of the IC _50_ cutoff points, when using both the 11-factor and DPPS encoding schemes.

**Table 4:** **11-factor and DPPS encoding for H2-IA**^**b**^

	**11-factor encoding**							
	***I*** ***C***_**50**_** cutoff**	**100**	**300**	**500**	**1000**	**5000**	**10000**	**15000**
	Sn Avg (%)	100.00	97.05	97.15	96.51	92.00	68.28	58.72
SR (*ℓ*_1_)	Sp Avg (%)	0.00	9.67	12.65	16.44	24.17	48.05	60.15
	Acc Avg (%)	90.00	81.82	79.41	73.58	62.38	57.49	59.60
	Sn Avg (%)	99.40	98.58	99.74	100.00	100.00	99.56	96.28
SVM	Sp Avg (%)	17.86	15.97	16.84	12.22	8.09	8.14	11.52
	Acc Avg (%)	91.94	84.11	82.32	74.84	59.80	50.82	45.31
	**DPPS encoding**							
	Sn Avg (%)	96.93	95.23	94.08	89.43	67.82	48.98	34.81
SR (*ℓ*_1_)	Sp Avg (%)	22.50	34.44	38.41	57.65	55.34	63.87	74.77
	Acc Avg (%)	89.99	84.63	82.40	79.84	62.39	56.94	58.79
	Sn Avg (%)	85.77	84.58	87.36	90.62	85.72	84.75	84.59
SVM	Sp Avg (%)	49.50	54.22	51.01	56.44	42.57	41.77	39.28
	Acc Avg (%)	82.40	79.26	79.77	80.15	66.88	61.81	57.30

**Table 5:** **11-factor and DPPS encoding for H2-IA**^**d**^

	**11-factor encoding**							
	***I*** ***C***_**50**_** cutoff**	**100**	**300**	**500**	**1000**	**5000**	**10000**	**15000**
	Sn Avg (%)	100.00	100.00	94.74	96.58	93.90	96.19	97.65
SR (*ℓ*_1_)	Sp Avg (%)	0.00	0.00	8.33	5.56	36.00	4.76	7.50
	Acc Avg (%)	90.00	80.00	75.80	77.00	70.48	70.22	68.80
	Sn Avg (%)	96.93	98.37	98.57	97.74	98.33	100.00	99.35
SVM	Sp Avg (%)	23.61	24.17	25.24	18.10	26.25	25.40	23.61
	Acc Avg (%)	86.19	85.80	83.17	79.80	69.33	78.94	75.00
	**DPPS encoding**							
	Sn Avg (%)	94.62	95.06	95.95	92.71	78.29	89.84	85.18
SR (*ℓ*_1_)	Sp Avg (%)	10.71	30.00	36.67	37.00	63.00	61.43	59.64
	Acc Avg (%)	82.68	83.46	83.51	80.27	72.23	81.82	77.02
	Sn Avg (%)	70.82	79.50	81.63	76.03	83.05	76.83	82.32
SVM	Sp Avg (%)	45.00	33.00	39.67	50.33	39.00	65.71	51.79
	Acc Avg (%)	67.33	71.39	72.98	70.08	65.30	73.73	72.63

**Table 6:** 11-factor and DPPS encoding for HLA-DPA1*0103/DPB1*0201

	**11-factor encoding**							
	***I*** ***C***_**50**_** cutoff**	**100**	**300**	**500**	**1000**	**5000**	**10000**	**15000**
	Sn Avg (%)	96.15	95.83	94.14	90.24	76.29	68.91	71.09
SR (*ℓ*_1_)	Sp Avg (%)	0.00	0.00	18.25	29.22	70.38	75.52	75.39
	Acc Avg (%)	86.21	79.31	77.28	71.29	73.31	72.64	73.61
	Sn Avg (%)	96.19	96.32	95.20	93.44	89.10	83.78	72.69
SVM	Sp Avg (%)	16.67	21.00	22.14	24.57	58.29	72.09	81.24
	Acc Avg (%)	88.02	82.83	78.72	72.21	73.59	77.06	77.69
	**DPPS encoding**							
	Sn Avg (%)	93.93	92.22	87.37	83.29	70.67	66.28	67.76
SR (*ℓ*_1_)	Sp Avg (%)	18.33	11.00	26.67	51.00	75.86	80.78	85.85
	Acc Avg (%)	85.82	77.69	73.64	73.30	73.31	74.67	78.44
	Sn Avg (%)	58.33	46.55	43.66	44.52	49.10	55.00	57.82
SVM	Sp Avg (%)	77.50	94.67	94.29	93.33	88.67	87.09	87.42
	Acc Avg (%)	60.42	55.14	55.08	59.72	68.99	73.62	75.33

**Table 7:** 11-factor and DPPS encoding for HLA-DRB1*0101

	**11-factor encoding**							
	***I*** ***C***_**50**_** cutoff**	**100**	**300**	**500**	**1000**	**5000**	**10000**	**15000**
	Sn Avg (%)	94.70	81.31	73.10	60.49	40.13	13.98	4.70
SR (*ℓ*_1_)	Sp Avg (%)	10.70	38.79	51.11	68.25	83.50	96.72	99.23
	Acc Avg (%)	59.31	57.55	59.42	66.00	76.65	87.98	91.74
	Sn Avg (%)	80.46	79.84	76.55	73.05	55.24	37.70	20.51
SVM	Sp Avg (%)	45.39	45.22	51.70	59.53	80.45	88.48	93.20
	Acc Avg (%)	65.67	65.24	62.66	64.64	76.46	83.11	87.43
	**DPPS encoding**							
	Sn Avg (%)	72.89	52.12	39.46	24.60	8.41	4.61	4.24
SR (*ℓ*_1_)	Sp Avg (%)	48.24	72.52	82.56	91.84	98.13	99.33	99.59
	Acc Avg (%)	62.51	63.52	66.27	72.36	83.96	89.32	92.03
	Sn Avg (%)	70.72	68.74	56.70	72.50	59.55	56.79	55.44
SVM	Sp Avg (%)	54.62	57.37	75.38	49.70	75.77	76.90	79.59
	Acc Avg (%)	63.94	62.38	68.32	56.32	73.20	74.78	77.67

**Table 8:** 11-factor and DPPS encoding for HLA-DRB1*0401

	**11-factor encoding**							
	***I*** ***C***_**50**_** cutoff**	**100**	**300**	**500**	**1000**	**5000**	**10000**	**15000**
	Sn Avg (%)	96.00	95.51	89.92	75.49	37.65	35.00	20.00
SR (*ℓ*_1_)	Sp Avg (%)	0.00	1.79	3.23	26.65	70.50	74.76	91.48
	Acc Avg (%)	77.42	69.47	61.44	53.55	58.37	61.94	82.26
	Sn Avg (%)	97.40	97.46	98.10	93.56	94.70	98.00	25.00
SVM	Sp Avg (%)	27.04	32.14	19.73	28.63	30.08	24.76	98.15
	Acc Avg (%)	84.28	78.95	72.30	64.45	53.86	48.39	86.45
	**DPPS encoding**							
	Sn Avg (%)	93.65	87.37	84.10	62.68	42.12	43.00	10.00
SR (*ℓ*_1_)	Sp Avg (%)	43.33	38.06	43.91	56.15	78.58	84.76	95.93
	Acc Avg (%)	84.23	73.53	70.94	59.70	65.18	71.29	84.84
	Sn Avg (%)	83.68	81.45	81.85	64.97	48.82	63.75	32.50
SVM	Sp Avg (%)	38.33	42.36	35.15	53.96	60.47	39.88	67.04
	Acc Avg (%)	75.11	70.40	66.33	60.00	56.26	47.58	62.58

## Discussion

Table 2 gives the results of our *ℓ*_1_-minimization algorithm and SVM predictions based on independent evaluation sets of three different epitope encoding methods. The experiments performed with our method revealed binder average accuracies in the range of 70-88*%* for the alleles used, similar to those predictive accuracies reported elsewhere [19].

**Figure 5:**
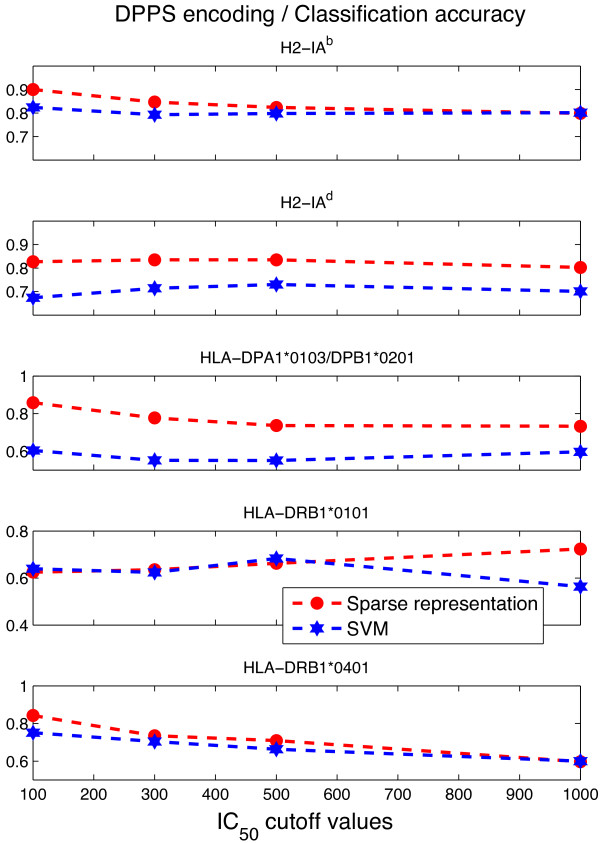
**DPPS encoding accuracy.** Comparison of predictive accuracy.

With DPPS encoding, the *ℓ*_1_-minimization method delivered higher AUC values of than any of the AUC of SVM for the same encoding scheme. However, with 11-factor encoding, the AUC obtained by SVM was higher than any AUC obtained by *ℓ*_1_-minimization. These results imply that different properties of amino acids are significant in the association process between the MHC-II molecule and the epitope, leading to higher performance in the prediction. This has a biological interpretation since nonbonding effects, such as electrostatic, van der Waals, hydrophobic interactions and hydrogen bond, play central roles in peptide-MHC interactions [14]. Hence, physico-chemical properties of amino acids should be considered when encoding epitopes for prediction. Since the *ℓ*_1_-minimization approach proposed here, where no model selection is involved, requires a more robust way of presenting the information to the algorithm, in this case, we conclude that the DPPS encoding is more appropriate. This is because DPPS directly relates to peptide-MHC association. In Figure 5 we show the accuracy of the sparse representation and SVM methods when working with DPPS encoding for different IC _50_ cutoff values. On the other hand, once the best choice of kernel has been obtained (model selection), SVM can handle less robust encoding schemes. We hypothesize that if more information is available in the encoding scheme of epitopes, our sparse representation algorithm could achieve higher performance.

## Conclusions

The proposed *ℓ*_1_-minimization algorithm is able to produce accurate classification of MHC class II epitopes with sensitivity, specificity and accuracy to those from SVM approaches. We studied the algorithm performance for peptide binding classification and compared it with SVM for a collection of both human and mice alleles. Our methodology relies on the natural selective nature of sparse representation in order to perform classification wherein no model selection is involved; with regards to robustness to outliers, our classification enabled us to discard bad training samples and handle noisy data [8,20]. This contrasts with the need to test different kernel functions in the SVM approach when trying to find the best separating hyperplane with a larger margin between classes. Our methodology involves a very simple learning stage and the use of an *ℓ*_1_-minimization solver first proposed in [8]. For the set of alleles studied in this work, we found the DPPS encoding scheme to be efficient in conjunction with the proposed methodology for peptide binding classification.

## Competing interests

The authors declare that they have no competing interests.

## Authors’ contributions

CAB conceived the idea of testing the algorithm for epitope prediction, gathered the data and prepared the datasets used during the research presented in the paper, and performed the experiments shown in the Results section. RSA conceived the algorithm proposed in the manuscript, wrote the computer programs implementing the method, and designed the figures interpreting the results. CL provided ideas about how to test the predictive accuracy of the algorithm. CAB, RSA, and CL collaborated in the writing of the manuscript, and read and approved the final version.
